# Complete Genome Sequence of Pseudomonas balearica Strain EC28, an Iron-Oxidizing Bacterium Isolated from Corroded Steel

**DOI:** 10.1128/MRA.00275-20

**Published:** 2020-05-07

**Authors:** Silvia J. Salgar-Chaparro, Genis Castillo-Villamizar, Anja Poehlein, Rolf Daniel, Laura L. Machuca

**Affiliations:** a Curtin Corrosion Centre, WA School of Mines: Minerals, Energy and Chemical Engineering, Curtin University, Bentley, Western Australia, Australia; b Genomic and Applied Microbiology & Göttingen Genomics Laboratory, Institute of Microbiology and Genetics, Georg-August University Göttingen, Göttingen, Germany; c Línea Tecnológica Biocorrosión, Corporación para la Investigación de la Corrosión C.I.C. Piedecuesta, Santander, Colombia; Loyola University Chicago

## Abstract

Pseudomonas balearica strain EC28 is an iron-oxidizing bacterium isolated from corroded steel at a floating production storage and offloading facility in Australia. Here, we report its complete genome sequence, which comprises 4,642,566 bp with a GC content of 64.43%. The genome harbors 4,164 predicted protein-encoding genes.

## ANNOUNCEMENT

Iron-oxidizing bacteria (IOB) derive energy from the oxidation of ferrous (Fe^2+^) to ferric (Fe^3+^) iron, resulting in the formation of dense iron oxide deposits that in contact with metals can promote under-deposit corrosion (1, 2). Several investigations have reported *Pseudomonas* spp. to be involved in microbiologically influenced corrosion (MIC) of different steels in industrial facilities and marine habitats (3–5). However, to our knowledge, the species Pseudomonas balearica has not been associated with MIC before. Pseudomonas balearica strain EC28 was isolated from corroded steel at a floating production storage and offloading (FPSO) facility in Australia. First, 1 g of corrosion products was scraped from the corroded steel and inoculated into 9 ml of anaerobic culture medium for IOB (6). The tube was incubated in darkness at 40°C for 28 days. Changes in the medium color and iron precipitation were observed after the incubation period. The culture was plated onto solid medium, which had the same IOB culture medium composition with the addition of 15 g/liter agar-agar. Plates were incubated under anaerobic conditions using anaerobic jars with AnaeroGen sachets (Oxoid, Thermo Fisher Scientific). Single colonies were subcultured and purified using the streak plate method. Pure colonies of strain EC28 showed iron precipitation, confirming its ability to oxidize ferrous iron.

Genomic DNA of *P. balearica* strain EC28 was extracted using the DNeasy PowerSoil kit (Qiagen, Hilden, Germany) following the manufacturer’s instructions. The isolated DNA was used to generate Illumina shotgun paired-end sequencing libraries. Sequencing was performed employing the MiSeq system and the MiSeq reagent kit v3 (600 cycles) as recommended by the manufacturer (Illumina, San Diego, CA, USA). In addition, DNA was prepared following the 1D genomic DNA by ligation (SQK-LSK109) protocol. Sequencing was conducted with a MinION device (Oxford Nanopore, Oxford, UK). End repair was performed using NEBNext formalin-fixed, paraffin-embedded (FFPE) repair mix (New England Biolabs, Ipswich, MA, USA). The library for Nanopore sequencing was loaded onto a SpotON flow cell Mk I (R9.4). The raw reads were quality filtered with fastp v0.19.4 (7). High-quality reads (1,717,952 short reads and 23,249 long reads) were hybrid *de novo* assembled using the Unicycler assembler v0.4.7 (8). Unicycler reported one circular chromosome of 4,642,566 bp (64.43% GC content, 124-fold coverage) and the absence of extrachromosomal elements. Bandage v0.8.1 (9) was used for visual validation of the assembly. Default parameters were used for all software unless otherwise specified.

The genome was annotated based on the NCBI Prokaryotic Genome Annotation Pipeline (10) v4.10. A total of 4,350 genes, including 4,164 protein-encoding genes with predicted functions, were detected. Moreover, 110 genes coding for hypothetical proteins, 60 tRNA genes, 12 rRNA genes, and 4 noncoding RNA (ncRNA) genes are also present. The average nucleotide identity (ANI) between EC28 and 27 publicly available genomes from different *Pseudomonas* species was determined with the Python module for average nucleotide identity analyses (pyANI) v0.2.7 (11). The ANI value above the threshold range (95 to 96%) of species delineation (12) with the genome of *P. balearica* strain DSM 6083 (GenBank accession number NZ_CP007511) indicates that strain EC28 belongs to the same species.

The genome of *P. balearica* strain EC28 contains a number of genes involved in iron metabolism, including iron uptake regulators, iron transporters, iron reductases, iron-binding proteins, and *c*-type cytochromes. Genes for complete dissimilatory nitrate reduction were also detected in the genome. The genome sequence provided here is expected to broaden our knowledge regarding corrosion processes initiated or accelerated by anaerobic nitrate-dependent iron oxidizers.

### Data availability.

The genome sequence of Pseudomonas balearica strain EC28 was submitted to GenBank under the accession number CP045858. The associated BioProject and BioSample accession numbers are PRJNA587695 and SAMN13198556, respectively. The raw reads have been deposited in the NCBI SRA database under the accession numbers SRR11483657 and SRR11483658.
